# Functional Orthodontic Treatment of Mandibular Condyle Fractures in Children and Adolescent Patients: An MRI Follow-Up

**DOI:** 10.3390/life12101596

**Published:** 2022-10-13

**Authors:** Hisham Sabbagh, Trayana Nikolova, Sara Carina Kakoschke, Andrea Wichelhaus, Tamara Katharina Kakoschke

**Affiliations:** 1 Department of Orthodontics and Dentofacial Orthopedics, University Hospital, LMU Munich, Goethestrasse 70, 80336 Munich, Germany; 2 Department of Radiology, University Hospital, LMU Munich, Marchioninistrasse 15, 81337 Munich, Germany; 3 Department of Oral and Maxillofacial Surgery and Facial Plastic Surgery, University Hospital, LMU Munich, Lindwurmstrasse 2a, 80337 Munich, Germany; 4 Department of General, Visceral, and Transplant Surgery, University Hospital, LMU Munich, Marchioninistrasse 15, 81337 Munich, Germany

**Keywords:** condylar fracture, pediatric fracture of the mandible, fracture treatment, TMJ, MRI, functional orthodontic treatment

## Abstract

Background: The purpose of this study was to retrospectively evaluate and follow up a conservative treatment approach with functional orthodontic appliances for the management of mandibular condyle fractures in children and adolescent patients. Methods: Between 2020 and 2022, the treatment records of patients with mandibular condyle fractures receiving a functional orthodontic treatment (FOT) were evaluated. In addition to the clinical and functional findings, magnetic resonance images of the mandibular condyles and surrounding structures were assessed. Results: Out of 61 patients, 8 met the inclusion criteria. The follow-up examination records showed no functional limitations. In 75% of cases, mild midline deviations persisted (mean 1.1 mm) without significant alterations to the occlusal relationships. Magnetic resonance imaging (MRI) showed the remodeling of the condyles and the restitution of the ramus heights, even in dislocated and displaced fractures. In three cases, a partial displacement of the articular disc was observed at the follow-up. No differences in the remodeling patterns were noted depending on age, sex, or fracture location. Conclusions: A FOT led to favorable functional and morphologic outcomes, supporting the concept of a conservative functional approach in children and adolescent patients. Functional adjunctive therapy should be considered in the conservative treatment of mandibular condyle fractures in growing patients.

## 1. Introduction

Mandibular fractures are among the most frequent facial fractures in children, and involve the mandibular condyles in 25% to 80% of cases [1,2,3,4,5]. Yet, the choice of the most appropriate treatment approach for fractures of the mandibular condyle in children and adolescents remains controversial [1,6,7,8,9,10,11,12]. Several study results or authors are in favor of a conservative treatment with [11,13,14,15,16,17,18,19,20] or without an orthodontic treatment [1,6,8,9,21] whilst others are in favor of a surgical open reduction and internal fixation [7,10,12]. Although there is a consensus that the pediatric condyle has a high regenerative capacity, it remains difficult to predict the extent of regeneration and remodeling in individual cases. If the remodeling capacity is overestimated and a conservative treatment is chosen, this may result in facial asymmetry, functional disorders, or the need for subsequent dysgnathic surgery [3,4,22]. In contrast, a primary surgical treatment carries the risk of facial nerve damage, scarring, growth disturbance, and other procedure-related as well as anesthesiological complications [21,23,24]. 

Various parameters such as age, type, and the location of fracture as well as the extent of fragment displacement or dislocation have been studied with regard to differential therapeutic considerations, but without leading to conclusive clinical guidelines. More recently, the effects of soft tissue injuries associated with condylar fractures on the remodeling ability and treatment outcomes have been investigated in magnetic resonance imaging (MRI) studies in adults and children [21,25,26]. Injuries to the disc and capsule of the temporomandibular joint (TMJ) have been found to contribute significantly to the development of complications, including traumatic ankylosis [17,26,27]. 

A recent systematic review concluded that the use of functional orthodontic appliances in growing patients may lead to an improved clinical rehabilitation through early controlled mobilization, restoring an organized functional condyle [28].

The aim of this study was to evaluate the clinical and morphological outcomes of a conservative treatment of mandibular condyle fractures in a group of children and adolescent patients that underwent a FOT.

## 2. Patients and Methods

This retrospective study was conducted at the LMU University Hospital and approved by the LMU Ethics Committee (Ref. No 21-0981). The inclusion criteria were: (1) age under 18; (2) isolated unilateral or bilateral condylar or subcondylar fractures; (3) a conservative FOT with a spring activator (no surgical treatment) between 2020 and 2022; (4) compliance with the follow-up and examination appointments and the completion of the FOT; and (5) the availability of MRI follow-up data. Patients with mandible fractures beyond the condyle or subcondylar region and panfacial fractures were excluded. 

**Figure 1:**
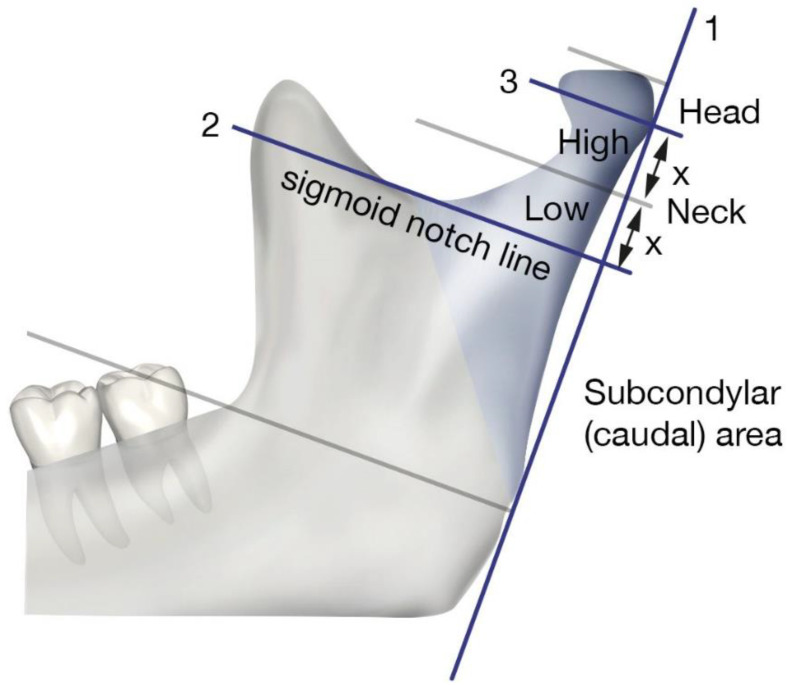
Classification of condylar fractures according to the AO-CMF trauma classification.

For all patients, a clinical examination for concomitant injuries was performed at the baseline, followed by a three-dimensional radiographic examination to confirm the fracture diagnosis. Condylar fractures were classified according to the AO-CMF trauma classification [29,30,31] (Figure 1).

**Figure 2:**
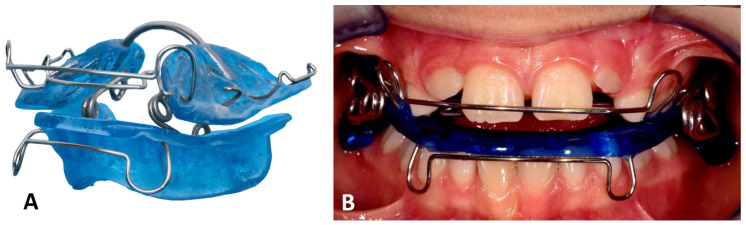
(**A**) Original spring activator for FOT designed to treat patients with open bite. (**B**) Clinically applied, modified spring activator with springs on the outside to facilitate insertion in cases of reduced mouth opening.

To enable mobilization, as soon as a painless mouth opening of more than 20 mm was possible, a maxillary and mandibular impression and bite registration were taken for the fabrication of a functional orthodontic appliance (a spring activator). The spring activator consisted of two plastic parts connected by loop springs, which determined the position of the upper and lower jaw in a clinically defined target position (Figure 2) [23,32,33]. Biomechanically, the spring elements of the device cause controlled mobility in the sense of a functional load on the temporomandibular joint whilst stabilizing the jaw relation and compensating for a loss of vertical height and tilting of the occlusal plane [23]. In addition, a soft diet for 10 weeks and daily wear of at least 16 h per day were prescribed to prevent the compression of the articular tissues [11,34]. The functional findings were documented at the time of the appliance insertion (timepoint *t_0_*).

Follow-up examinations were performed every six to eight weeks; in each case, recording the functional findings and adjusting the appliance if necessary. Adjustments to the appliance served to restore the correct fit and retention as well as the activity of the spring mechanism. The FOT was completed after 9 months.

The functional and clinical findings included mouth opening (maximum incisal edge distance), midline deviations in the centric occlusion and during mouth opening, the extent of excursive movements (laterotrusion and protrusion), the palpation of the joints and masticatory muscles, joint sounds, and the occlusal relation [35].

Magnetic resonance images were obtained with a 3 Tesla MRI system (Magnetom Ingenia und Phlips dStream Flex MR-coil 10 cm, Philips Healthcare, NL) using proton density-weighted images (PDW-SPIR; slice thickness: 1.5 mm; total acquisition time: 13:20 min; repetition time: 2330 ms; echo time: 25 ms; spin echo 288 × 288). The radiological assessment included an evaluation of the condyles, the articular disc, and signs of soft tissue injury. The physiologic position of the disc was assumed by the posterior band of the disc at the superior (12 o’clock) position relative to the top of the condyle in the glenoid fossa in the closed-mouth position [34]. Joint effusion was defined as an area of high intensity on the T2-weighted images [36,37]. Disc deformities were defined as alterations to the physiologic biconcave shape of the disc [36,37]. The evaluation was performed using imaging software (Visage Imaging Inc., USA) by a radiology resident (T.N.) and reviewed by a board-certified radiologist (with 8 years of experience).

## 3. Results

### 3.1. Patient Collective and Fracture Classification

The treatment records of 61 patients with mandibular condyle fractures between 2020 and 2022 were screened for eligibility. Fourteen patients received a FOT and eight patients (six male, two female) met the inclusion criteria. The age range of the included patients at the time of trauma was between five and fourteen years (mean age 8.3 years) (Table 1). Three patients showed bilateral condylar fractures and five patients showed unilateral fractures. The most frequent type of fractures were condylar neck fractures (n = 6), followed by condylar head fractures (n = 3) and subcondylar fractures (n = 2). The fractures were all dislocated and in six cases the head fragment was displaced (cases (1), (2), (3), (4), (7), and (8)). Except in one case (1), all fractures were accompanied by vertical height loss and angulation. Fragmentation was present in four cases ((1), (4), (7), and (8)). In two cases, the condylar head fragment was distorted ((6) and (7)). A specific level 3 condylar process system code [30] was applied to the analysis of the initial 3D images (cone beam computed tomography (CBCT) or computed tomography (CT)).

### 3.2. Functional Findings

At the initial examination (t_0_), all patients exhibited a restricted mouth opening of less than 30 mm (mean 22.8 mm) and a reduced range of mandibular movements (Table 2). A physiologic range of mandibular protrusion and laterotrusion movements of 6.0 mm or more was assumed [2]. Midline deviations during the mouth opening and in the centric occlusion were also present in all included cases, although the pre-existence of midline deviations before the trauma could not be excluded. The midline deviations ranged from 1.5 mm to 4.0 mm (mean 3.1 mm). Joint sounds such as clicking or crepitation were recorded in two cases. Pain on palpation of the temporomandibular joints was noted more frequently (n = 7) than on the masticatory muscles (n = 2). Alterations to the occlusal relationships such as an anterior crossbite (case (3)), a lateral crossbite (cases (5) and (8)), or an open bite (case (6)) were present in four cases.

At the final examination (timepoint 1, t_1_), no patient had a restricted mouth opening (mean 45.3 mm) or a reduced range of mandibular movements and there was no case of joint ankylosis (Table 2). Pain symptoms on palpation were not reported. Joint sounds were recorded in two cases, but only one case involved the same individual as at t_0_. Midline deviations persisted in 75.0% of patients in a range of 0.5–2.0 mm (mean 1.1 mm). Alterations to the occlusal relationships did not persist after the FOT.

### 3.3. Radiological Findings

**Figure 3:**
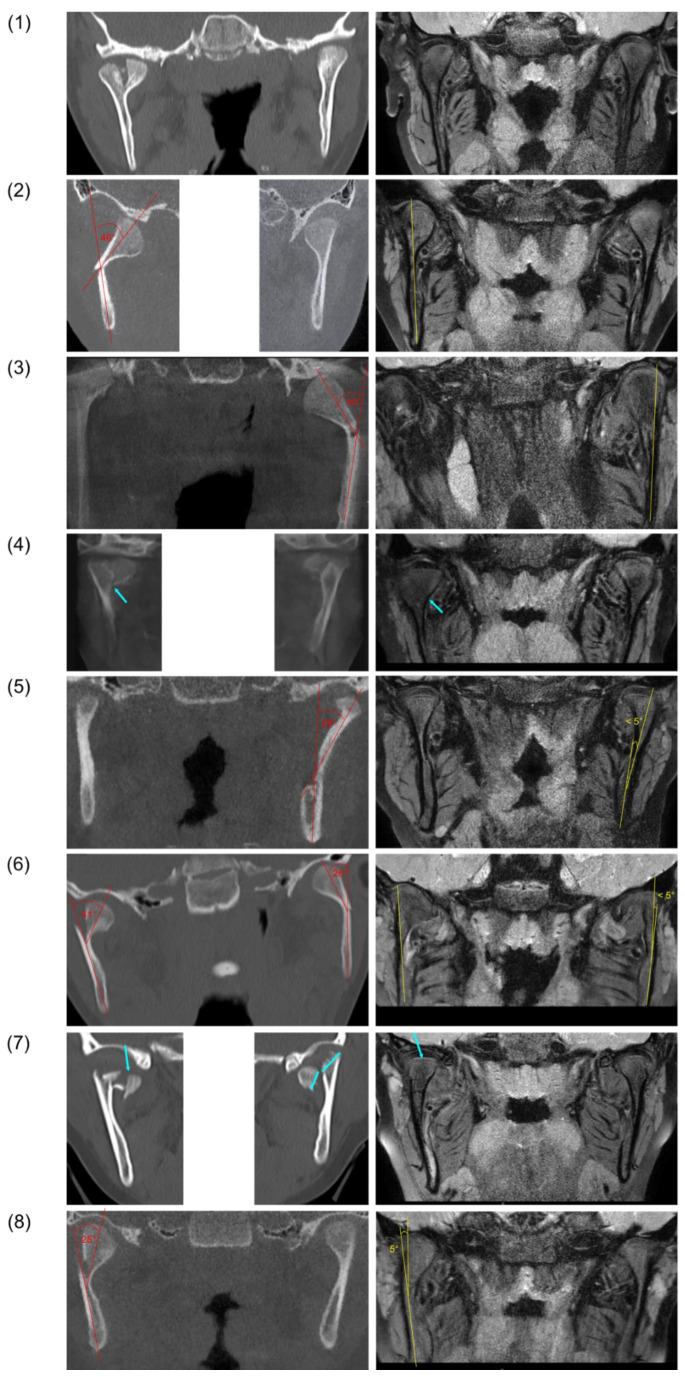
Pretreatment CBCT/CT images (left) and follow-up magnetic resonance images (right) after fractures of the mandibular condyle and FOT in eight patients (rows 1–8). Angulation between fracture fragments is indicated in red (pretreatment) and yellow (follow-up). Fracture lines and bony scar lesions are marked by blue arrows.

**Figure 4:**
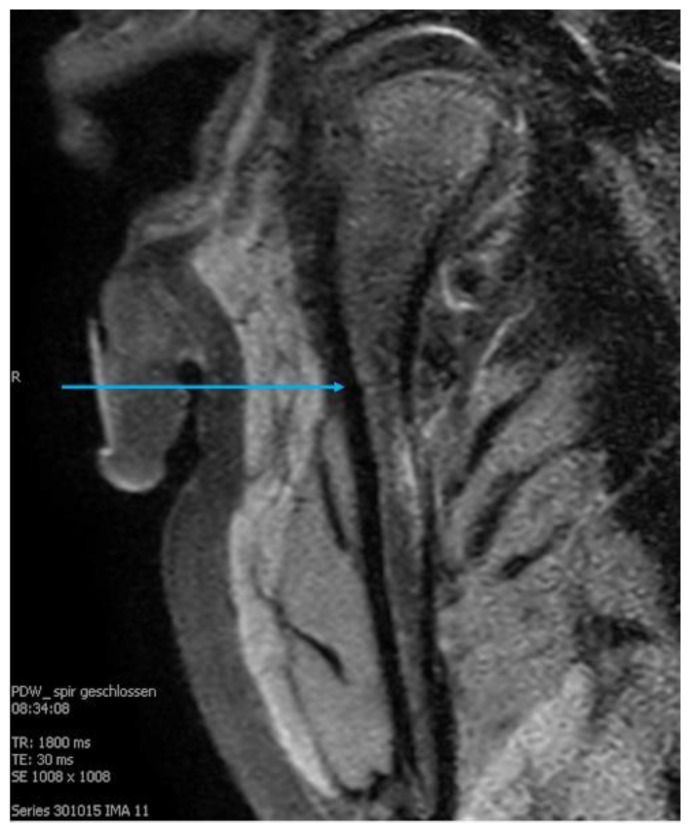
Magnetic resonance image of a mandibular condyle during follow-up (2). A line in the sense of a bone scar between the former fracture fragments is visible (blue arrow).

**Figure 5:**
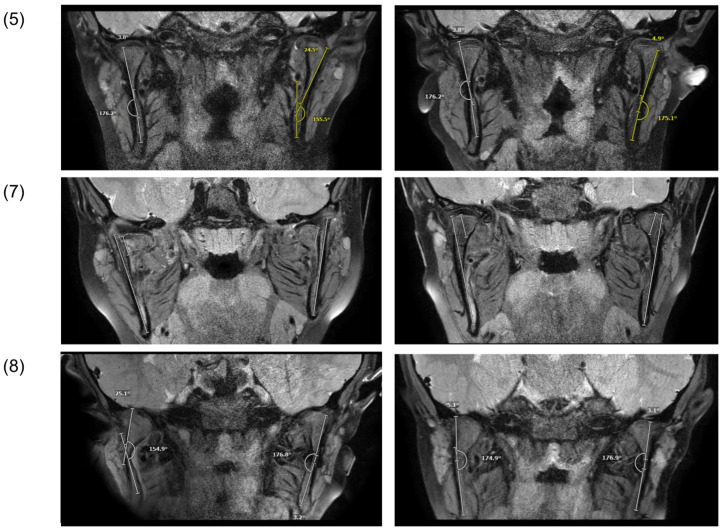
Magnetic resonance image of patients after trauma (t_0_, left) and after treatment (t_1_, right).

Pretreatment CBCT/CT images and MRI follow-up data were available for the eight included patients (Figure 3). After the treatment (t_1_), a remodeling of the mandibular condyle could be observed in all cases. The remodeled condyles showed no morphological irregularities in shape, or a slightly broader shape in the area of the condylar neck (Figure 3; (1) and (8)). Angulated condylar heads showed a full uprighting at the follow-up (Figure 3; (2), (3), (5), (6), and (8)). No significant shortening of the ramus heights was observed where assessable. In three cases, bony scar lesions were identified in the area of the pretreatment fracture line between the segments (Figure 3; (4) and (7) and Figure 4; (2)).

The articular disc showed a partial displacement in three cases (Figure 3; (2), (5), and (8)) whereas in the other cases, a physiological position of the disc was observed. Joint effusion or disc perforation were not observed at t_1_. 

On the MRI images, the former fracture site could still be identified as a scar lesion after 9 months of treatment (Figure 4).

Pretreatment MRI images were additionally available in three cases: (1), (7), and (8) (Figure 5). After the trauma (t_0_), reduced ramus heights and angulation of condylar fragments were evident. In one case (Figure 5; (7)), the retrodiscal attachment was torn and the left condylar fragment and disc were medially displaced outside the fossa. After the treatment (t_1_), no morphologic differences could be observed in comparison with the contralateral side.

## 4. Discussion

The functional orthodontic treatment of condylar fractures in children and adolescent patients showed favorable clinical, functional, and radiologic outcomes and resulted in the functional rehabilitation of the TMJ in all of the consecutive included cases.

No pain symptoms or limited mandibular mobility were noted during the follow-up examinations after the end of the treatment. In two cases, joint sounds in the form of clicking remained due to a disc displacement with a reduction. Mild midline deviations persisted in 75% of cases; however, with a mean of 1.1 mm, the observed deviations were minor. The occlusal relationships showed no significant alterations such as anterior or lateral crossbites or an open bite.

Interestingly, in contrast to previous studies that concluded that a conservative treatment in adolescent patients yields clinically and functionally good outcomes but does not necessarily restore the integrity of the articular process and thus the ramus height [9,38,39], the uprighting and healing of the fracture segments could be documented in the present study by MRI. No differences in the remodeling results were noted with respect to age, sex, or fracture location. In one case (7), a severely displaced fracture showed complete restitution even after an initial vertical height loss of more than 9 mm and an angulation of the fragment of more than 25°. The initially fully displaced disc showed only a partial displacement at the end of the treatment, although the retrodiscal attachment was torn. 

The processes of condylar healing and remodeling remain poorly understood [39]. In growing patients, remodeling appears to lead to a normal anatomy in favorable cases whereas in other cases, irregular patterns with changes in the condyle position, condylar angles, and shapes are the result [8,11,20,40]. Bifid condyles—so-called V-shaped alterations and hyperplastic bone formations, among others—are reported in the literature [40,41]. The reasons for these different outcomes have mostly been attributed to the severity of the trauma, type, and location of the fracture and the age of the patients [7]. Therefore, attempts have repeatedly been made to define the treatment indications based on age limits or fracture types, which is further complicated by the various different classifications [42,43,44,45,46,47] that coexist at an international level [31]. In the position paper derived from the International Bone Research Association (IBRA) Symposium, which evaluated the treatment strategies for mandibular condyle fractures, a consensus was reported from a panel of experts that preferred a non-surgical treatment in the first five to six years of age [7]. In contrast, no consensus could be reached for the treatment of patients between six and twelve years of age, and other clinical guidelines are also not available [7]. These attempts are based on reasonable assumptions as both the severity of the trauma and the structural biology of the juvenile condyle with the cell-rich proliferation layer containing prechondroblasts have a significant impact on the prognosis [48]. Yet, given the conflicting recommendations and reported outcomes in the literature, these do not appear to be the only determining factors. Conservative treatment approaches are often grouped under the collective terms “closed treatment” or “non-surgical treatment” and methods for a functional adjunctive therapy are rarely considered, although there is increasing evidence of improved treatment outcomes [28,48]. 

Functional orthodontic appliances used for the treatment of condylar fractures have been well-described in the literature [11,13,14,16,18,19,20,23,49,50]. However, the spring activator appliance used in this study could be particularly suitable compared with other rigid or elastic appliances [23]. Biomechanically, the effect of the loop springs placed dorsally on the first molars results in an inversion of motion, causing a controlled distraction in the articular area instead of compression during temporal and masseter muscle activity [23]. With the spring activator, the mobilization and simultaneous stabilization of the occlusal plane and jaw relationship can be achieved and the loss of vertical ramus height can be compensated for. These conditions may provide a more favorable environment for remodeling according to Moss’s functional matrix theory [51,52,53,54]. However, the results of our study demonstrate healing not only in terms of remodeling, which is regarded as resorption in the displaced direction and bone regeneration in the original position [6,18,55,56], but also in terms of reduction and fracture healing, a phenomenon previously disputed by other groups of authors [9,38,39]. As documented with magnetic resonance imaging, it seems possible to redirect the displaced condylar fragment and the articular disc toward the former position and restore the original ramus height.

Continuous advancements in surgical techniques, including improved osteosynthesis allowing the stable fixation of fracture fragments and the early mobilization of the TMJ, have improved the outcomes of the open reduction and internal fixation (ORIF) approach and have led to increasing popularity and use for the treatment of condylar fractures in children and adolescent patients [7,21,57,58]. However, the ORIF approach carries the risk of severe complications such as facial nerve damage, scarring, wound infection, and other complications [21,57,59]. Furthermore, multiple surgical interventions under general anesthesia with the corresponding risks are required in the majority of cases, either for the removal of the MMF due to incompliance, or when the removal of the osteosynthesis material is advised [60]. Lastly, functional problems and compromised anatomical positioning after ORIF are not uncommon [3,61]. 

Taking these considerations into account, differential therapeutic decisions remain in the hands of the clinician, who must weigh the risks and benefits of the different treatment modalities. When choosing the conservative therapeutic approach, a functional adjunctive therapy should be considered. 

## 5. Strengths and Limitations

Although condylar fractures are relatively common injuries, it is difficult to recruit a homogeneous patient population and to comprehensively document the course of treatment and follow-up [9]. After a severe trauma involving facial fractures, patients usually present to an oral and maxillofacial surgery clinic or other surgical centers and a referral for a functional orthodontic treatment is only rarely considered, even if a non-surgical treatment is chosen. This is problematic because usually only small numbers of cases are available for the scientific evaluation of the functional orthodontic treatment method or the documentation is often inadequate in the more extensive studies that have been conducted [28]. In the present study, the case number was also too small to draw general conclusions or to compare outcomes with other treatment modalities. Rather, the present study raises questions on the current understanding of condylar fracture healing and provides information about an established treatment protocol with favorable outcomes. Furthermore, the need for the differentiation of a conservative treatment with and without a functional adjunctive therapy is emphasized and a methodology for a morphologic follow-up by MRI is presented. 

To date, morphologic changes have been studied mostly with conventional plain radiographs and CT scans, which do not reveal soft tissue changes to the TMJ [21,37] and expose children and adolescents to avoidable radiation. In view of the associations between soft tissue injuries and the remodeling capacity [21,25,26], follow-up examinations of condylar fractures using MRI seem more appropriate. With the high sensitivity of MRI, even bony scar lesions can be made visible and bone healing can be monitored.

In this context, a follow-up of approximately 12 months after the trauma may be considered to be sufficient to follow the remodeling process, which has been found to be usually completed within three to six months, depending on the location and type of fracture [6,62]. Although the further growth of patients was not followed, other studies have shown that long-term complications such as growth disturbances are rare even in the presence of radiographic aberrations, given a functionally positive outcome of the initial healing [38,41,55,62]. 

## 6. Conclusions

A functional orthodontic treatment led to favorable clinical, functional, and morphologic outcomes, supporting the concept of a non-surgical functional approach in children and adolescent patients. In addition to the remodeling processes, the reduction and healing of dislocated fracture segments and the articular disc were also documented. A functional adjunctive therapy should be considered in the conservative treatment of mandibular condyle fractures in growing patients. 

## Figures and Tables

**Table 1:** Summary of the characteristics of included patients.

Case	Sex	Age at Trauma	Type of Trauma/Etiology	Fracture Side	Type of Fracture AO-CMF	Specific Level 3 Condylar Process System Code [30]										
						Location	Fragmentation	Vertical Apposition	Sidewards Displacement	Angulation	Displacement Head Fragment/Fossa	Displacement Caudal Fragment/Fossa	Distortion of Head	Loss of Ramus Height	Angulation in °	Height Loss in mm
(1)	M	7 years, 5 months	Bicycle	Right	Condylar head fracture	M	0	0	-	-	1, a	-	0	0	-	0 mm
(2)	M	5 years, 6 months	Bicycle	Right	Low condylar neck fracture	-	0	-	1	2, m	1, a, m	0	0	1	46°	3.2 mm
(3)	M	14 years, 9 months	Fall	Left	Low condylar neck fracture	-	0	-	0	1, m	1, m	0	0	1	40°	5 mm
(4)	F	7 years, 3 months	Scooter	Bilateral	Right: condylar head fracture	M	0	0	-	-	0	-	0	0	-	0 mm
					Left: condylar head fracture	M	1	0	-	-	0	-	0	0	-	0 mm
(5)	M	10 years, 7 months	Play	Left	Subcondylar fracture	-	0	-	1	1, l	1	0	0	1	25°	4.5 mm
(6)	F	8 years, 10 months	Play	Bilateral	Right: high condylar neck fracture	-	0	-	1	1, m	0	0	0	1	41°	4 mm
					Left: low condylar neck fracture	-	0	-	0	1, m	0	0	0	1	26°	2 mm
(7)	M	8 years, 6 months	Bicycle	Bilateral	Right: condylar head fracture	P	1	2	-	-	1, a	-	1	1	-	5.5 mm
					Left: condylar head fracture	P	2	1	-	-	1, a, m	-	1	1	-	9 mm
(8)	M	5 years, 7 months	Fall	Right	Subcondylar fracture	-	0	-	1	1, m	0	0	0	1	25°	3 mm

**Table 2:** Functional and clinical findings at Timepoint 0 (t_0_, before treatment) and Timepoint 1 (t_1_, after treatment).

Sign	No. Patients (*t*_0_)	No. Patients (*t*_1_)	% (*t*_1_)
Reduced range of mouth opening	8/8	0/8	0
Reduced range of lateral movement	8/8	0/8	0
Reduced range of protrusion	8/8	0/8	0
Arthralgia	7/8	0/8	0
Myalgia	2/8	0/8	0
Joint sounds (clicking or crepitation)	2/8	2/8	25.0
Mandibular midline deviation during mouth opening	8/8	6/8	75.0
Mandibular midline deviation in centric occlusion	8/8	6/8	75.0
Alteration to occlusal relationship (crossbite or open bite)	4/8	0/8	0

## Data Availability

Not applicable.
